# The subacute toxicity and underlying mechanisms of biomimetic mesoporous polydopamine nanoparticles

**DOI:** 10.1186/s12989-023-00548-4

**Published:** 2023-10-08

**Authors:** Bang-Yao Chen, Si-Ying Hong, Han-Min Wang, Yi Shi, Peng Wang, Xiao-Juan Wang, Qian-Yang Jiang, Ke-Da Yang, Wei Chen, Xiao-Ling Xu

**Affiliations:** 1 Shulan International Medical College, Zhejiang Shuren University, 8 Shuren Street, Hangzhou, 310015 China; 2 ICU, Longhua Hospital, Shanghai University of Traditional Chinese Medicine, 725 South WanPing Road, Shanghai, 200032 China; 3 Department of Clinical Pharmacy, The First Affiliated Hospital, School of Medicine, Zhejiang University, 79 Qingchun Road, Hangzhou, 310003 PR China

**Keywords:** Mesoporous polydopamine, Systematic evaluation, Subacute toxicity, Metabolite, Gut microbiota

## Abstract

Recently, mesoporous nanomaterials with widespread applications have attracted great interest in the field of drug delivery due to their unique structure and good physiochemical properties. As a biomimetic nanomaterial, mesoporous polydopamine (MPDA) possesses both a superior nature and good compatibility, endowing it with good clinical transformation prospects compared with other inorganic mesoporous nanocarriers. However, the subacute toxicity and underlying mechanisms of biomimetic mesoporous polydopamine nanoparticles remain uncertain. Herein, we prepared MPDAs by a soft template method and evaluated their primary physiochemical properties and metabolite toxicity, as well as potential mechanisms. The results demonstrated that MPDA injection at low (3.61 mg/kg) and medium doses (10.87 mg/kg) did not significantly change the body weight, organ index or routine blood parameters. In contrast, high-dose MPDA injection (78.57 mg/kg) is associated with disturbances in the gut microbiota, activation of inflammatory pathways through the abnormal metabolism of bile acids and unsaturated fatty acids, and potential oxidative stress injury. In sum, the MPDA dose applied should be controlled during the treatment. This study first provides a systematic evaluation of metabolite toxicity and related mechanisms for MPDA-based nanoparticles, filling the gap between their research and clinical transformation as a drug delivery nanoplatform.

**Supplementary Information:**

The online version contains supplementary material available at 10.1186/s12989-023-00548-4.

## Introduction

With the booming development of nanotechnology, porous nanomaterials have become a hot topic in the field of drug delivery due to their unique structure and good physicochemical properties [1]. For example, mesoporous silica, silver, gold, and manganese dioxide nanoparticles have been widely studied for their homogeneous and adjustable mesopore pore size [2–4], stable skeleton structure, large specific surface area and modifiable inner surface [5]. However, a large number of studies have demonstrated that the fabrication of porous nanomaterials remains complicated with multistep operations, including template preparation, template-directed synthesis, and template removal. Too many steps are prone to the generation of secondary synthesis products and residual organic reagents. They are not only potentially damaging to the environment but also have some acute or subacute toxicity to the organism. Thus, there is an urgent imperative to develop biomimetic nanomaterials that combine simplicity of fabrication, environmentally friendly biodegradation, and improved biocompatibility.

Drawing inspiration from mussels, Jing Tang et al. [6] successfully synthesized biomimetic mesoporous polydopamine (MPDA) nanoparticles. They employed the high molecular weight block copolymer, polystyrene-block-poly (ethylene oxide) or PS-b-PEO, as a soft template. In mildly alkaline conditions (pH ~ 8.5), dopamine (DA) underwent spontaneous oxidation, and was deposited on the surface of self-assembled PS-b-PEO micelles [7]. This synthesis process yielded nanoscale particles with a dimension of approximately 200 nm and a mesoporous structure approximately 16 nm in diameter. These aggregates exhibited augmented stability in near-neutral pH conditions (7 − 11) compared to strongly alkaline conditions (pH > 11) [8]. In the creation of mesoporous structures in PDA, hard and soft templating methods are utilized. While the hard templating method allows greater control over nanomaterial morphology, it’s complex, time-consuming, and unsuitable for mass production. Alternatively, soft templates provide simpler construction, diverse morphology, and cause less environmental pollution, though they encounter issues such as low yields and limited availability of template agents.

The surfaces and mesopores of MPDA nanoparticles abound with phenyl, amino, and hydroxyl groups. These features endow them the superior capability to load various chemical drugs, such as DOX (doxorubicin) and SN38 (7-ethyl-10-hydroxycamptothecin), via mechanisms like π-π stacking and/or hydrogen bond interactions [9]. Additionally, MPDA particles have unique physicochemical properties, including metal ion chelation, photothermal conversion, environmental (pH or laser)-triggered drug release, high chemical reactivity and facile modification capabilities. Upon incorporating a targeting moiety, these nanoparticles can achieve targeted delivery to specific lesions, making MPDA-based nanodevices a focus of intensive studies for various therapeutic applications, including cancer theranostics, antibacterial strategies, and antifibrotic therapies.

In terms of biosafety, MPDA have shown great biocompatibility with various cells, and no organ changes were detected in rats post-injection [10]. However, the processes by which MPDA is metabolized or degraded in vivo, as well as the potential toxicity of its degradation products or metabolites, remain poorly understood. Existing research suggested that the pH-responsive properties of PDA could facilitate its degradation at lower pH levels, a typical characteristic of tumor microenvironments [11]. Furthermore, PDA-based nanoparticles are also reportedly degradable by H_2_O_2_ and free radicals present in the body. Moreover, Jin et al. [12, 13] identified PDA degradation products in the human acute monocytic leukemia cell line THP-1 using high-performance liquid chromatography (HPLC) and speculated that the most dominant compounds in PDA degradation products were dopamine and some intermediate products, such as quinine and PDA segments. None of these components affected cell viability, reaffirming PDA compatibility. Given the existing knowledge gap regarding full biocompatibility, there is a pressing need for systematic investigation into the subacute toxicity and underlying mechanisms of biomimetic mesoporous polydopamine nanoparticles [4].

In this regard, MPDAs were prepared by the soft template method, and different doses of MPDAs were injected into the tail vein of healthy male mice in this study. After 7 days of treatment, the changes in body weight, organ index, pathology and routine blood tests were analyzed to evaluate subacute toxicity. The possible mechanism was determined based on the microbiome and metabolomics of intestinal content and metabolomics measurements for serum. This study will supplement the toxicity data of MPDA and provide a theoretical basis for its clinical translation.

## Materials and methods

### Materials

Dopamine hydrochloride, doxorubicin hydrochloride and ammonia (NH_3_·H_2_O, 25–28%) were purchased from Shanghai Macklin Biochemical Technology Co. Limited (Shanghai, China). NH_2_-PEG-NH_2_ (Mw = 5000) was purchased from Shanghai ToYongBio Technology Co., Ltd. (Shanghai, China). Pluronic F127, Pluronic 123, and 1,3,5-trimethylbenzene (TMB) were provided by Sigma Chemical Co. (St. Louis, MO). All other chemicals used were of analytical or chromatographic grade.

The mice distributed by Institute of Cancer Research (ICR mice) aged 6–8 weeks and weighing 20 g were obtained from Shanghai Silaike Laboratory Animal Limited Liability Company, and given adequate food and water. The animal experiments were conducted according to the National Institutes of Health (NIH, USA) guidelines for the care and use of laboratory animals, with surgical procedures approved by the Committee for Animal Experiments of Longhua Hospital Affiliated to Shanghai University of Traditional Chinese Medicine (PZSHUTCM2212120003).

### Preparation of mesoporous polydopamine

Biomimetic mesoporous polydopamine (MPDA) nanoparticles were prepared by the soft template method as previously reported [14]. Briefly, P-123 (0.030 g), F-127 (0.075 g), and DA (0.15 g) were uniformly dispersed in 40% ethanol solution (20 mL) under the condition of water bath ultrasound (BC-3B, Shanghai Benting Instrument, China). After that, TMB (0.4 mL) was added to the above solution and sonicated for 10 min in a 40% probe ultrasonic instrument (XM-1500T, Xiaomei ultrasonic instrument, China) at a frequency of 3 s and intermittent 2 s. Then, MPDA nanoparticles were obtained by adding ammonia water (0.375 mL) to the mixed solution and mechanically stirring for 4 h at room temperature. To remove the excess water-soluble agents, the mixed solution was separated in a centrifuge (9500 rpm, 5 °C, 15 min). Then, the precipitates were dispersed in ultrapure water with sonication. The washing process was repeated twice. The crude products were further dispersed in absolute ethanol and washed three times. Subsequently, 0.05 g of NH_2_-PEG-NH_2_ was added to the prepared nano-system above and subjected to magnetic stirring for 8 h to ensure that MPDA was adequately modified by NH_2_-PEG-NH_2_. This modification serves to enhance the stability of MPDA nanoparticles. Finally, PEG-modified MPDA was obtained by centrifugation. All tests concerning MPDA nanoparticles in this paper refer to those that are PEG-functionalized.

### Size distribution and morphology

The size distribution of MPDA nanoparticles was analyzed using dynamic light scattering (DLS) with a Zetasizer from Malvern Co., UK. Morphological examinations were carried out using transmission electron microscopy (TEM) with a JEOL JEM-1230 from Japan.

### Photothermal effect

#### Laser irradiation of MPDA at different concentrations under the same power

One milliliter of aqueous solutions of MPDA nanomaterials with different concentrations (1 mg/mL, 0.2 mg/mL, 0.25 mg/mL, 0.125 mg/mL, 0.625 mg/mL, and purified water) were placed in a 96-well plate at room temperature (24 °C). The nanomaterials were laser irradiated for 300 s with a fixed power (1.68 A, 1.5 W) of the laser irradiator. Afterwards, the temperature of the aqueous solution of MPDA nanomaterials was measured by the thermometer at an interval of 30 s each time (n = 5).

#### Laser irradiation of MPDA at the same concentration under different powers

Four aliquots of MPDA nanomaterial aqueous solution (1.5 mg/mL, 1 mL) were placed in a 96-well plate at room temperature (24 °C). The nanomaterials were laser irradiated for 300 s with a fixed power (1.68 A 1.5 W, 0.54 A 0.33 W, 0.75 A 0.5 W, 1.31 A 1 W, 1.86 A 1.5 W) of the laser irradiator. Afterwards, the temperature of the aqueous solution of the MPDA nanomaterial was measured by the thermometer at an interval of 30 s each time (n = 5).

#### Thermal stability experiment of MPDA

MPDA nanomaterials (1.5 mg/mL, 1 mL) were placed into a 96-well plate at room temperature (24 °C). After that, the laser irradiated the nanomaterials with the laser irradiator (laser wavelength 808 nm, laser spot 0.09 mm^2^) at fixed power (1.68 A, 1.5 W). Then, the temperature of the dispersion naturally returned to room temperature. The operation was repeated twice. The temperature of the aqueous solution of MPDA nanomaterials after irradiation was measured by the thermometer at an interval of 30 s each time (n = 3).

### Drug loading experiment

To study the drug loading property of MPDA nanomaterials, doxorubicin hydrochloride (1 mg/mL, 1 mL) was added to different concentrations of MPDA nanomaterials (0.05 mg/mL 1 mL, 0.1 mg/mL 1 mL, 0.2 mg/mL 1 mL). The mixture was stirred overnight and then centrifuged (9500 rpm, 15 min). After centrifugation, 100 µL of the supernatant was added dropwise to a black shaded 96-well plate, and the absorbance was measured by a fluorescence photometer (SpectraMax i3x, Molecular Devices, USA, doxorubicin hydrochloride, Ex = 504 nm, Em = 550 nm). To prepare the standard curve, aqueous solutions of doxorubicin hydrochloride at different concentrations (0.1 mg/mL, 0.2 mg/ml, 0.4 mg/mL, 0.8 mg/mL, 1 mg/mL) were diluted and measured by a fluorescence photometer (SpectraMax i3x, Molecular Devices, USA, doxorubicin hydrochloride, Ex = 504 nm, Em = 550 nm).

The drug loading rate of MPDA nanomaterials was calculated according to the following formula:

$$Drug{\text{ }}loading{\text{ }}rate{\text{ }} = \left( {{C_{DOX@}}_{MPDA} - {C_{\sup ernate}}} \right)/{C_{DOX@}}_{MPDA} \times 100\%$$

$$Encapsulation{\text{ }}efficiency = \left( {{m_{feeding{\text{ }}DOX}}{\text{ - }}{C_{\sup ernate}}\,V} \right)/{m_{feeding{\text{ }}DOX}} \times 100\%$$

### Toxicity experiments with MPDA

#### Body weight

Twenty-eight mice were randomly divided into 4 groups (n = 7). The initial body weights of the mice were recorded before the experiment, and subsequently, the mice were given saline (control group), 78.57 mg/kg MPDA (H-MPDA group), 10.87 mg/kg MPDA (M-MPDA group), and 3.61 mg/kg MPDA (L-MPDA group) by tail vein injection. Mice were weighed every 2 days after administration to assess MPDA nanoparticle toxicity.

The dosage calculation process was listed as below.

1. Based on the drug dosage, the feeding ratio of drug and MPDA, as well as the mice bodyweight documented by previous studies (Table 1), we were able to calculate the amount of MPDA to administer to each mouse. Our calculations determined that the lowest amount required was 0.1 mg (low group) or 0.3 mg (median group). In our study, the mice in the median group had a bodyweight of 27.5875 g, while those in the low group weighed 27.6625 g. Accordingly, the dosage for the low group was set at 3.61 mg/kg, while that for the median group was set at 10.87 mg/kg.
2. The dosage for the high group was determined based on the saturated solubility of MPDA as prepared in our research. This was done with the intention of evaluating whether administering MPDA at the highest concentration would cause any acute or subacute damage to the body. It is worth noting that this dosage falls well below the maximum dosage given in previous treatment.

**Table 1:** The amount of PDA given in previous studies

PDA dosage	Drug dosage	Feeding ratio of drug and PDA	Mice bodyweight	Amount of PDA	References
10 mg/kg			15–17 g	0.15–0.17 mg	[15]
12 mg/kg			18–20 g	0.22–0.24 mg	[16]
	5 mg/kg	0.25:1 (w:w)	16 g	0.32 mg	[17]
	5 mg/kg	1:2.5 (w:w)		0.25 mg per 20 g bodyweight	[18, 19]
	10 mg/kg	1:5 (w:w)	25 g	1.25 mg	[20]
	10 mg/kg	0.25:1, 0.5:1, 1:1 and 2:1 (w:w)		0.8 mg per 20 g bodyweight	[21]
	2.5 mg/kg	5:45(w:w)	21-25 g	0.45–0.56 mg	[22]

#### Organ index

On the seventh day after administration, all mice were weighed and anesthetized. All tissues, including the heart, liver, spleen, lungs, kidneys, and colon, were harvested. The removed organs were carefully placed in Phosphate-Buffered Saline (PBS) solution, and excess connective tissue was removed from the organs with surgical scissors and weighed. The organ index was calculated by the following equation.

$$Organ{\text{ }}index{\text{ }}\left( \% \right) = {\text{ }}{W_1}/{W_2} \times 100\%$$

W_1_ is the weight of the organ, and W_2_ is the body weight of the corresponding mouse.

#### Routine blood tests

At the end of the tests, 1 mL of peripheral blood was collected and analyzed using a fully automated hematology analyzer (TEK8500 VET, TECOM, CHN).

#### Examination of pathological sections

The tissues and organs of mice were fixed with 4% formaldehyde for 48 h and then dehydrated with ethanol solutions at different concentrations of 30%, 50%, 70%, and 90%. The fixed tissues were cleared and embedded in paraffin wax with xylene, sectioned with a microtome (4 μm thick), dewaxed, and finally stained with hematoxylin-eosin. Finally, the morphological changes in the main organs after staining were observed by light microscopy.

#### Immunohistochemical staining

Liver tissue sections, previously deparaffinized and rehydrated, were subjected to a blocking procedure in blocking buffer for a duration of 30 min at room temperature. Following this, these sections were exposed to antibodies specifically designed to interact with ATP-binding cassette subfamily B member 11 (ABCB11). Subsequently, these sections were subjected to incubation with secondary antibodies conjugated with horseradish peroxidase (HRP), these antibodies were rabbit-derived and held specific reactivity against mouse IgG. Finally, the expression level of ABCB11 in the livers after staining were observed by light microscopy.

#### Gut microbiota sequencing and analysis

The genomic DNA of bacterial communities in mouse colon contents was extracted and subjected to 16 S rRNA gene sequencing on the Illumina HiSeq 2500 (PE250) platform by Tinygene Biotechnology Co., Ltd. in Shanghai, China. The obtained reads were analyzed using Usearch version 7.1 to cluster operational taxonomic units (OTUs) at a 97% similarity threshold. Bioinformatics analysis was performed at the OTU level, with alpha diversity indexes such as Chao1, Shannon, and Simpson calculated using Mothur version 1.39.5. For beta diversity analysis, principal component analysis (PCA) and principal coordinate analysis (PCoA) were conducted in R language. Differential analysis was performed using LEfSe software, which employed linear discriminant analysis (LDA) to estimate the impact of species abundance on different conditions.

#### Liquid chromatography‒mass spectrometry (LC‒MS)-based metabolomics

##### LC‒MS metabolomic examination of feces

Prior to execution, mice were placed in dry, clean cages, and fresh feces were collected from each group and allowed to stand in an ice-water mixture for 1 h. Samples of feces (0.05 g) were placed in a 1.5 mL centrifuge tube, 80% methanol (0.8 mL) was added, and the sample was ground at 65 Hz for 90 s and vortexed for 30 min at 4 °C. The sample was then allowed to stand for 1 h at -40 °C, vortexed for 30 s, and allowed to stand for 0.5 h at -40 °C. The supernatant was then centrifuged (12,000 rpm, 4 °C, 15 min) in a centrifuge tube and allowed to stand at -40 °C for 1 h. The centrifugation was repeated, and the supernatant was mixed with dichlorophenylalanine (0.14 mg/mL, 5 µL) and transferred to the injection vial. Detection and analysis were performed by LC‒MS (Waters, UPLC; Thermo, Q Exactive). Metabolite profiles were subjected to multivariate statistical analysis, specifically principal component analysis (PCA) and partial least squares discriminant analysis (PLS-DA), to identify differences between groups. Hierarchical clustering analysis (HCA) and metabolite correlation analysis were performed to explore the relationships between metabolites. To interpret the biological significance of the metabolites, we conducted metabolic pathway and functional analyses.

##### LC‒MS metabolomic examination of mouse serum

Serum stored at -80 °C was taken, and serum samples were processed as described in 2.6.6.1 and subjected to the same statistical analysis.

### Statistical analysis

Comparative analysis of differences between groups was calculated by one-way analysis of variance (ANOVA) with SPSS 19.0 (95% confidence interval). A significant difference was set at ***p < 0.001, **p < 0.01, *p < 0.05. Values are displayed in the form of Mean ± SD.

## Results

### The preparation and characterization of MPDA nanoparticles

#### Size distribution and morphology

**Fig. 1:**
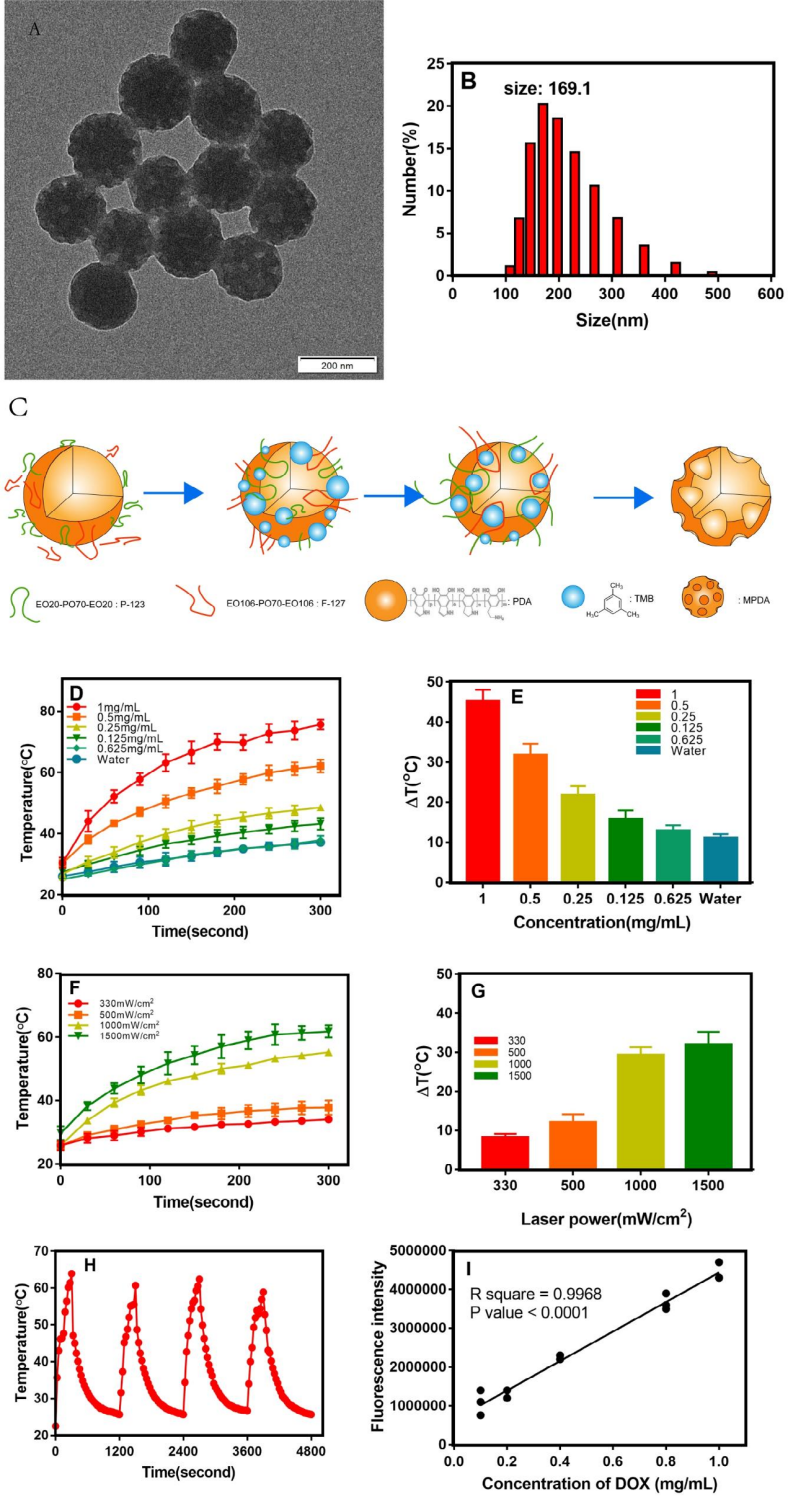
The characterization of MPDA nanoparticles. **(A)** TEM images for as-prepared MPDA particles. Scale bar = 200 μm. **(B)** The diameter of the MPDAs measured using DLS. **(C)** Synthetic routes of the preparation of MPDA nanoparticles. **(D, E)** Photothermal conversion and the improving temperature of MPDAs at different concentrations under 808 nm laser irradiation (1.5 W/cm^2^). **(F, G)** Photothermal conversion and the improving temperature of MPDAs (1 mg/mL) under 808 nm laser irradiation with different exposure intensity (0.33–1.5 W/cm^2^). **(J)** Photothermal stability study of MPDAs during four circles of heating-cooling processes. **(l)** Linear relationship between Fluorescence signal intensities and concentrations of DOX.

Understanding the physicochemical properties of MPDA are preconditions for its toxicity testing. Since various research of MPDA-based nanoplatforms focus on tumor treatment [23], we initially prepared MPDA and further investigated the general parameters to ensure its potential application for tumor therapy. MPDA nanoparticles were synthesized by the soft template method according to our previous report [24]. DA easily self-aggregates to form mesoporous-structured PDA in an alkaline environment with the addition of F-127, P-123, and TMB as templates. After preparation, PEG was engineered onto the surface of MPDA to improve the stability. As shown in the TEM images (Fig. 1A), the MPDA nanoparticles showed a spherical shape, and the average particle size of the MPDA nanoparticles was 169.1 ± 12.8 nm (Fig. 1B). The porous structure on the surface of the particles can be clearly observed in the high-precision TEM image, and the average pore size is estimated at 48.77 ± 15.86 nm. Moreover, the MPDA-based nanoparticles maintained a stable particle size over the course of 7-day storage (Figure S1), and exhibited minimal drug release behavior with ultimately only a 5% release (Figure S2). The synthetic routes of the preparation of MPDA nanoparticles was shown in Scheme Fig. 1C.

#### Photothermal effect

To confirm the photothermal conversion efficiency of MPDA, a near-infrared laser was employed (808 nm, 1.5 W, 5 min). Under laser irradiation, the temperature of MPDA rapidly rose, showing concentration-dependent behavior. The mean temperature of the 0.0625 mg/mL MPDA group (ΔT ≈ 12.9 ℃) in 5 min was almost the same as that of pure water (ΔT ≈ 11.1℃). (Fig. 1-D, E) In contrast, MPDA at the concentration of 1 mg/mL demonstrated a more significant temperature (ΔT ≈ 45.3 ℃) when compared with other groups (0.5 mg/mL: ΔT ≈ 31.8 ℃, 0.25 mg/mL: ΔT ≈ 21.8 ℃). The photothermal conversion rate of the 1 mg/mL MPDA dispersion can be obtained as 17.9%.

In addition, the photothermal effect of the MPDA aqueous solution is positively correlated with the laser irradiation intensity (Fig. 1F, E) and duration. Notably, the MPDA dispersion did not show degradation after four cycles of heating and cooling by continuous laser irradiation, exhibiting excellent photostability and thermal stability (Fig. 1H). Hence, MPDA can be used in phototherapy (PPT) due to its relatively high photothermal conversion rate, which is frequently used as a photothermal agent to kill tumor cells.

#### Drug encapsulation efficiency

The encapsulation of DOX into MPDA nanoparticles was prepared by simply mixing the chemotherapeutic drug with the MPDA dispersion overnight. As displayed in Table 2, the encapsulation efficiency of DOX decreased as the feeding ratio of DOX to MPDA increased. It reached the highest level (98.99 ± 0.53%) when the ratio was 25%.

**Table 2:** Drug loading and encapsulation efficiency of MPDA at different drug administration ratios

DOX : MPDA Dosing ratio	1:1	1:2	1:4
EE	79.53$\pm$1.24%	88.63$\pm$2.59%	98.99$\pm$0.53%
DL	55.16$\pm$4.62%	37.05$\pm$2.31%	24.27$\pm$0.23%

### Pathological examination in mice

#### Body weight changes

**Fig. 2:**
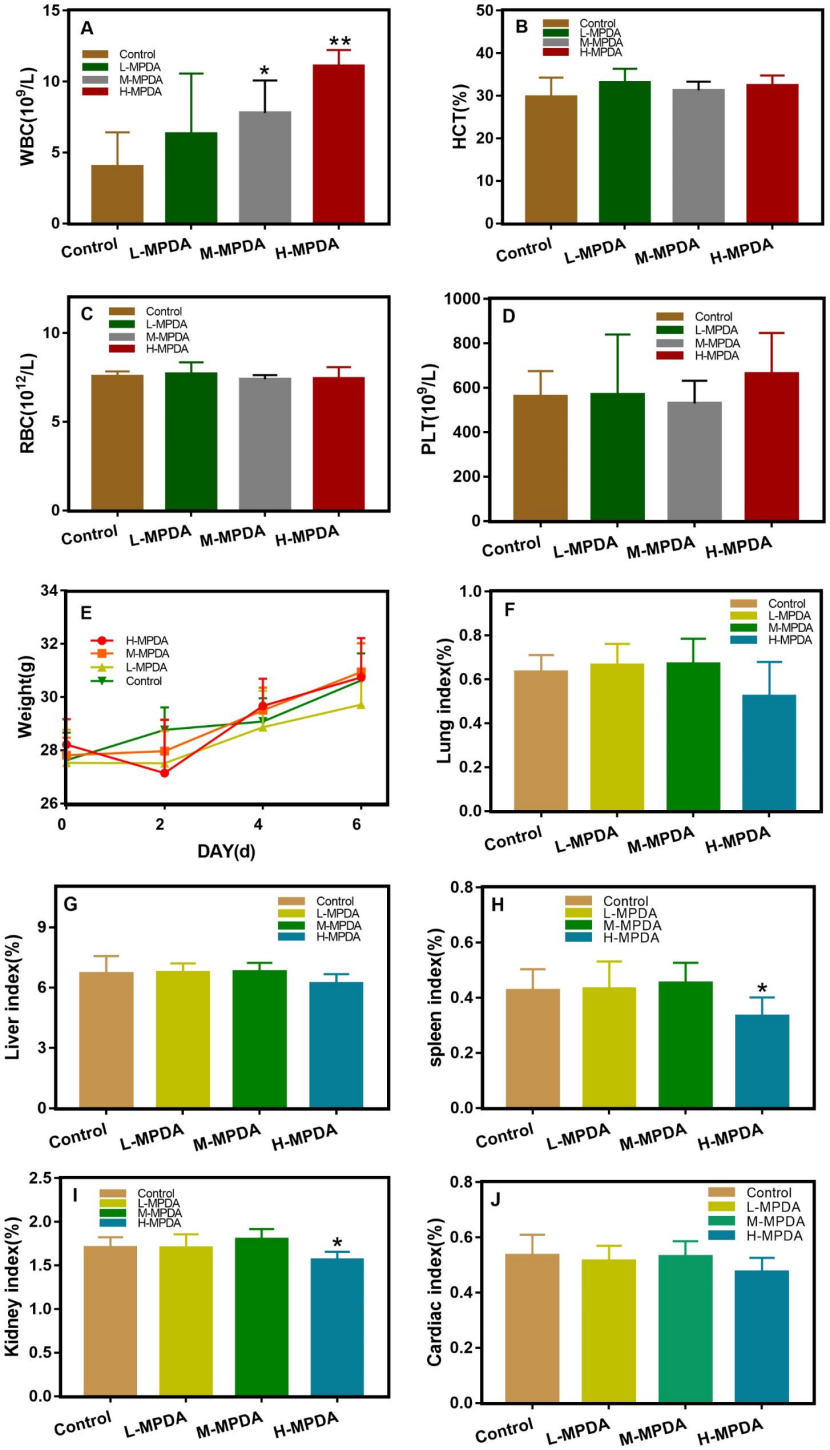
The in vivo toxicity of MPDA nanoparticles. **(A, B, C, D)** The performance of various blood indexes under different concentrations of MPDAs-treated groups (78.57 mg/kg MPDA, 10.87 mg/kg MPDA, and 3.61 mg/kg MPDA). **(E)** Line plot of body weight change from day 2 to day 6 in each MPDAs treatment group. **(F, G, H, I, J)** The organ index of heart, liver, spleen, lung, and kidney, ** *p* < 0.01, * *p* < 0.05 compared with Control group

After tail vein injection, the mice did not show obvious symptoms such as dyspnea and difficulty in movement, while the bodyweight of different dose-treated groups decreased to different degrees within 2 days after injection (Fig. 2E). The decline within 2 days was particularly pronounced in the high-dose group, which may be related to acute stimulation in terms of invasively exogenous nanocarriers. Subsequently, the body weight of mice gradually returned to the normal value in each dose group and maintained a stable increase, which implies that MPDA dispersion did not interfere with the growth rate of mice in the long term.

#### Routine blood examination

Despite having little effect on body weight, nanoparticles may have adverse effects on hematological parameters. All hematological parameters except white blood cell (WBC) count were not significantly different between the MPDA-injected group and the control group at all injection doses (Fig. 2A - D). Notably, a slight increase in WBCs was observed in the H-MPDA group, indicating that a high dose of MPDA injection should be monitored for the immune response.

#### Changes in the organ index

The organ indexes were mainly detected in the heart, liver, spleen, kidney, and lung. As shown in Fig. 2F - J, no significantly different changes in the organ index were observed except in the spleen, and kidney. Consistent with the WBC results, the H-MPDA group exhibited a significantly lower spleen index than the other dose groups. It is well known that the spleen is an immune organ, and the spleen index depends on the number of proliferating lymphocytes. Therefore, the immune system function of H-MPDA-treated mice may be partially damaged.

As for the kidney changes, it may be that the high dose of MPDA injection caused a certain renal burden, which caused significant kidney decline in the H-MPDA-treated mice. Review of related studies revealed that the most frequently utilized dosage for MPDA nanoparticles typically falls at the median level (10.87 mg/kg). This conventional dosage level has not demonstrated any significant toxicity, suggesting that the benefits likely outweigh any negligible safety risks at lower to median dosage levels. However, our research raises potential safety concerns with regard to kidney and spleen functions when administering the maximum dosage. Therefore, our findings strongly recommend that high-dose applications of MPDA (78.57 mg/kg) should involve meticulous monitoring and management to uphold a satisfactory risk-benefit ratio.

#### Pathological changes in organs

**Fig. 3:**
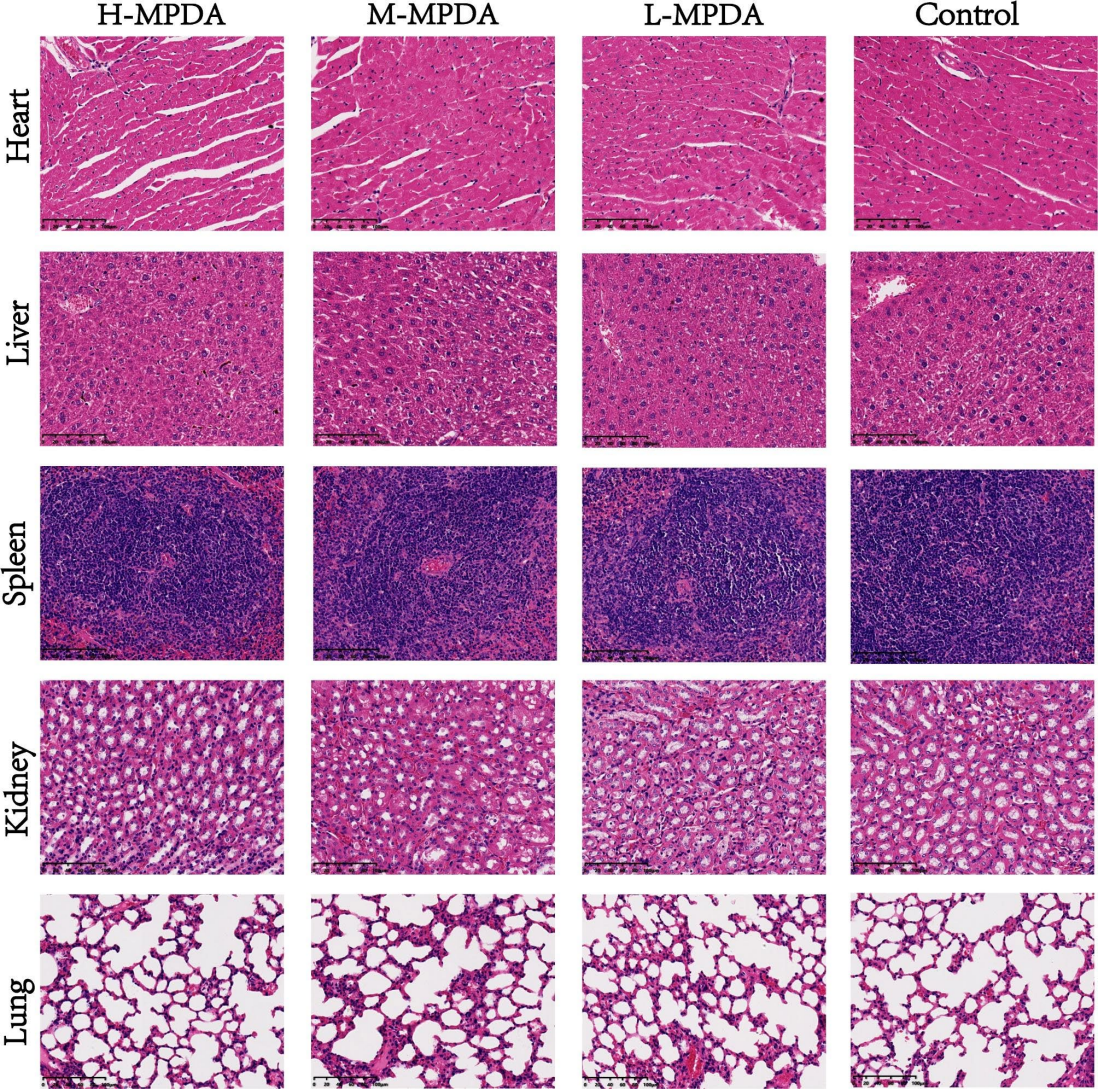
Histopathology images of heart, liver, spleen, lung, and kidney collected from the H-MPDA treated group, M-MPDA treated group, L-MPDA treated group, and Control group. Scale bar = 100 μm

Given changes in response to immunity, histological examination was further performed. As shown in Fig. 3, no signs of inflammation or pathological changes were observed in the liver and spleen, although massive evidence indicated that MPDA was primarily distributed in the liver and spleen. Importantly, analysis of the spleen, including the white pulp, red pulp and marginal zone, did not show any histopathological changes, suggesting the absence of spleen dysfunction. It was reported that a large quantity of nanoparticles accumulated in the lung can lead to inflammation, granuloma formation, and lung dysfunction; however, no lung histological changes were found in our study. No histopathological abnormalities were observed in other tissues, including the heart and lung.

High doses of MPDA might instigate immune activation or affect the genesis and release of leukocytes, thereby leading to an abundant production of white blood cells in peripheral circulation. This increase in leukocyte count could be discerned during standard hematological examination. This heightened state of immune activation could necessitate a substantial expenditure of energy and biosynthetic materials, resulting in potential utilization or depletion of splenic reserves. These reserves may comprise vast quantities of immunomolecules, such as immunoglobulins and cytokines, and the mobilization of immune cells, thereby contributing to a reduction in spleen mass. However, it’s plausible that no overt pathological modifications are discernible in histological preparations, potentially attributable to the inability of such influences to elicit significant morphological modifications within a brief timeframe. An alternate explanation could be that high-dose MPDA primarily impacts hematological constituents or elicits alterations at a cellular level. These intricacies may not be readily observable at the tissue level due to their microstructural nature.

### Effect of MPDA on gut microbiota in mice

#### OTU clustering results

**Fig. 4:**
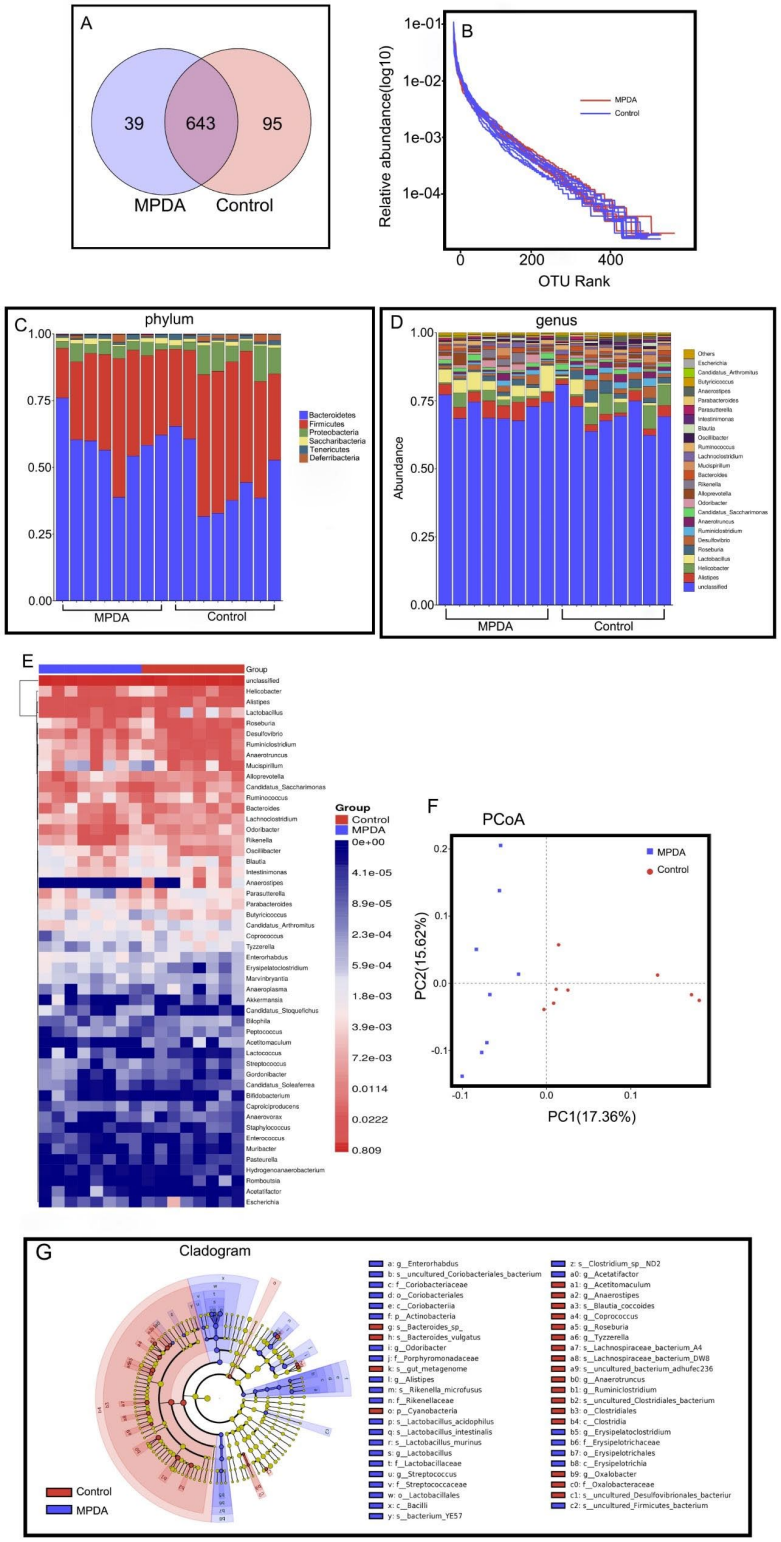
Effect of MPDA on gut microbiota in mice. **(A)** The Venn diagram of Control group and MPDA groups. **(B)** The rank abundance curve of Control group and MPDA groups, **(C, D)** The bar chart of species distribution at the phylum and genus level. **(E)** The heatmap of species distribution at the genus level. **(F)** The PCoA map of Control group and MPDA groups. **(G)** Linear discriminant analysis effect size plot of Control group and MPDA groups

Through VENN analysis of operational taxa (OTUs), the similarity and overlap of OTU composition between different treatment groups were studied. As shown in Fig. 4A, the Venn diagram can represent the common OTUs and unique OTUs of each sample. The common OTUs of the control group and MPDA group were 643, while the OTUs of the control group and MPDA group were 95 and 39, respectively, indicating that MPDA intervention could affect the composition of OTUs in the intestinal flora of mice.

#### Species complexity analysis

The rank abundance curve, with the OTU rank as the abscissa, and curve width reflects species composition abundance, with a wider curve width representing higher species composition abundance [25]. The relative abundance of OTUs was the ordinate, which can visually reflect the abundance and evenness of the species in the sample. Rank-abundance distribution curves account for both species abundance and distribution uniformity. As shown in Fig. 4B, the horizontal width of the curve in this study is large and relatively flat, indicating that the abundance and distribution uniformity of microbial species in this study are reasonable and reliable.

#### Effect on the α diversity of intestinal microorganisms

The diversity and richness of intestinal microbial communities in mice were evaluated using the Shannon and Simpson indexes for diversity, and the Chao and ACE indexes for richness. Comparing the control group to the MPDA group, the Shannon, and Simpson indexes were significantly higher in the control group, while the ACE and Chao indexes were significantly higher as well. These results suggest that MPDA at a certain dosage had an impact on the α diversity of the intestinal microbiota in mouse colon, leading to significant changes in both diversity and richness when compared to the control treatment.

#### Effect on β-diversity of intestinal microorganisms

To evaluate the impact of MPDA on the β diversity of intestinal microbiota, we conducted sample-level cluster analysis and principal coordinate analysis (PCoA). The PCoA map depicts the similarity of species composition structure between samples, with closer samples indicating higher community structure similarity and clustering together. As evidenced in Fig. 4F, the PCoA results based on the relative abundance of OTUs clearly differentiated the samples from each group. The control group samples were mainly clustered together in a compact distribution with high similarity, while the MPDA group samples were concentrated in different regions, suggesting that MPDA exposure disrupts the intestinal microbiota of mice. These findings were consistent with the α diversity analysis and suggest that MPDA may have some impact on the original order of intestinal flora in mice.

#### Identification of different species of bacteria

Linear discriminant analysis effect size plot (LEfSe, *p* < 0.05, LDA > 2) analysis was performed to obtain significantly different bacteria. As a result, a total of 49 significantly different bacteria were found at the genus level (Fig. 4G), and the genera with higher relative abundance were comparatively analyzed. The results suggest that MPDA may cause a certain degree of toxicity to the body by causing disorder of the flora.

##### Phylum level

At the phylum level, the intestinal microbes of the mice in this study mainly included Bacteroidetes, Firmicutes, Proteobacteria, Saccharibacteria, Tenericutes, Deferribacteres, Cyanobacteria, Actinobacteria, and Verrucomicrobia. Among them, Bacteroidetes and Firmicutes were the dominant flora in the MPDA group and the control group (Fig. 4C). The sum of their relative abundances was 90.55%; however, the proportion of the two dominant phyla in each sample was different. Compared with the control group, Bacteroidetes (58.26%, 45.48% in the control group), Firmicutes (34.19%, 43.17% in the control group), Deferribacteres (0.61%, in the control group), and the relative abundance of Cyanobacteria (0.78% and 0.24% in the control group) had a significant change (*p* < 0.05).

Studies on the ratio of Bacteroidetes to Firmicutes (Bac/Fir) have shown that the ratio is widely considered to have an important effect on the maintenance of normal intestinal homeostasis, and changes in the ratio may indicate microecological imbalance [26]. The Bac/Fir ratio in the MPDA group was 1.70 compared with 1.05 in the control group, indicating a significant increase in the intestinal microbiota of mice (*p* < 0.05), which means that the MPDA could change the structure of the intestinal flora of mice. It has been suggested that an increased Bac/Fir ratio may contribute to obesity and intestinal inflammation [27], which is consistent with our conclusion. In a study based on Deferribacteres, which is a bacterium that can obtain energy through obligate or facultative anaerobic metabolism, the iron metabolism of Deferribacteres in the gut microbiota is associated with intestinal iron balance, where abnormal iron metabolism occurs [28]. Thus, it can cause the occurrence and development of intestinal tumors. At the same time, Cyanobacteria is also a microorganism in the human gut that plays an important role in the host’s metabolism and disease risk. Some scholars recently analyzed the fecal samples of 25 healthy infants and compared them with the samples collected from infants with acute gastroenteritis. Members of the Cyanobacteria phylum had a higher abundance in the intestines of infants with viral diarrhea compared with healthy controls in the study. This study suggests that intestinal Cyanobacteria may be associated with gastrointestinal diseases. Accordingly, we can speculate that MPDA may induce and change the gut microbiota in mice and be detrimental to mice. In Fig. 4E, it is evident that the administration of MPDA via tail vein injection significantly reduced the abundance of Anaerostipes bacteria compared to the Control group. According to studies, Anaerostipes bacteria are known to convert inositol into short-chain fatty acids (SCFA), which play an important role as potential probiotics in maintaining gut balance. Therefore, the significant decrease in Anaerostipes bacteria can be considered as potential evidence for the presence of certain subacute toxicity associated with MPDA.

##### The level of genus

The 51 most abundant genera were selected, clustered at both the sample and microbial species levels based on their abundance in each sample, and included in the heatmap. (Fig. 4D, E) The top four dominant species in the control group were Helicobacter ganmani, Mucispirillum sp. 69, [Clostridium] leptum and Bacteroides acidifaciens. After MPDA intervention, the order of dominant bacteria was changed, and Lactobacillus_acidophilus became the main bacteria, followed by Helicobacter_ganmani and Clostridiales_bacterium_CIEAF_020.

A growing number of studies have shown that liver diseases are associated with disorders of the intestinal microbiome. Gut dysbiosis is characterized by a significant increase in opportunistic pathogens represented by Enterobacteriaceae. A streptozotocin-high fat diet (STZ-HFD)-induced NASH-HCC C57BL/6J mouse model, which is highly related to human hepatopathy, has been used to find significant structural changes in gut microbiota during hepatopathy progression. Atopobium spp., Bacteroides spp., Bacteroides vulgatus, and Bacteroides_acidifaciens play an important role in the pathogenesis of hepatopathy [29]. In this study, the abundance of Lactobacillus_acidophilus was significantly increased after tail vein injection of MPDA, which is consistent with the after mouse fecal metabonomics study of bile metabolism markers in this paper.

### Fecal metabolomics study

#### Multivariate analysis of LC‒MS data

**Fig. 5:**
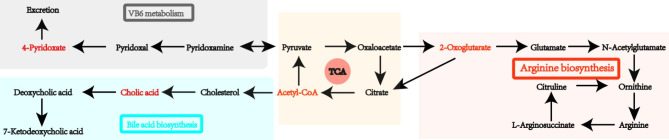
The potential network of metabolic pathways

Raw data were collected by ultrahigh-performance liquid chromatography (Acquity, Waters, USA) and high-resolution mass spectrometry (Q Exactive, Thermo Fisher Scientific, USA). Then, principal component analysis (PCA) (Fig. 5A, B) was used for dimensionality reduction analysis, and an orthogonal projection to latent structures squares-discriminant analysis (OPLS-DA) model was used to analyze the difference in endogenous substances between the two groups. The more similar the functional composition of the samples in the PCA, the closer they are in the PCA plot. In this study, the control samples were basically concentrated in one area, with a relatively dense distribution and high similarity. According to the PCA conclusion, there was an obvious separation between the MPDA group and the control group, indicating that the endogenous substances in the feces of the experimental mice had changed. Compared with the control group, the spots in the MPDA group were scattered in different areas, which means that MPDA can interfere with the intestinal microenvironment of the experimental mice and may affect the original orderly intestinal flora. According to the results of OPLS-DA, the correlation between the MPDA group and the control group could be clearly distinguished. In POS mode, the evaluation index was R2Y = 0.991, Q2 = 0.796, and in NEG mode, the evaluation index was R2Y = 0.998, Q2 = 0.789. Both R2 and Q2 were parameters of the OPLS-DA model, which could represent the interpretation and prediction ability of the model. The closer R2Y and Q2 are to 1, the more stable and reliable the model is. In this study, R2 is greater than 0.9, and Q2 is greater than 0.7, which indicates that the OPLS-DA model is stable and reliable. It can also be seen that there is a significant difference between the MPDA group and the control group, from which it is inferred that there is an obvious change in fecal metabolites in the MPDA group, as shown in Fig. 5C, D.

#### Screening and identification of potential biomarkers

Two parameters, variable influence on projection (VIP) and P value, were used to screen differential metabolites. Potential biomarkers with significant contributions were screened by an S-plot map (VIP > 1 and *p* < 0.05) (Fig. 5E, F). The potential biomarkers were initially identified based on their accurate relative molecular masses and mass spectrometry analysis results, matched with the Human Metabolome Database (HMDB) database, and validated by MS/MS. Finally, the differential metabolites between the control and MPDA groups were selected, as shown in Table 3. As shown in Fig. 5G, H, we screened significant markers and established Receiver Operating Characteristic (ROC) curves to evaluate their reliability. Taking palmitoleic acid and arachidonic acid as examples, the results showed that the area under the curve (AUC) was 0.889 and 0.972, respectively, which indicated that the model had excellent recognition performance in the MPDA group and the control group, and the model was reasonable and reliable.

**Table 3:** The differential metabolites between the control and MPDA groups

Ionization mode	Identification		Formula	RT (min)	Measured Mass (m/z)	MPDA	Control	Pathway
ESI-	N-Acetylneuraminic acid		C_11_ H_19_ N O_9_	0.792	308.0987	↓**	↑**	Arginine biosynthesis
	Inosine		C_10_ H_12_ N_4_ O_5_	1.468	267.0734		↑*	Purine metabolism
	adrenosterone		C_19_ H_24_ O_3_	7.314	299.1653	↓**	↑**	Steroid hormone biosynthesis
	Arachidonic acid		C_20_ H_32_ O_2_	12.716	303.2328	↑**	↓**	Biosynthesis of unsaturated fatty acids
	Eicosapentaenoic acid		C_20_ H_30_ O_2_	12.12	301.2171	↑**	↓**	Biosynthesis of unsaturated fatty acids
	Estriol		C_18_ H_24_ O_3_	6.948	333.1709	↓*	↑**	Steroid hormone biosynthesis
	Uridine		C_9_ H_12_ N_2_ O_6_	1.223	243.0621	↓**	↑**	Pyrimidine metabolism
	alpha-Linolenic acid		C_18_ H_30_ O_2_	12.218	277.2171	↑**	↓**	alpha-Linolenic acid metabolism, Biosynthesis of unsaturated fatty acids
	Cholic acid		C_24_ H_40_ O_5_	7.043	407.2799	↓**	↑**	Primary bile acid biosynthesis
	Guanosine monophosphate		C_10_ H_14_ N_5_ O_8_ P	1.438	362.0494	↑**	↓**	Purine metabolism
	4-Pyridoxic acid		C_8_ H_9_ N O_4_	1.65	182.0451	↓**	↑**	Vitamin B6 metabolism
	N-Acetylglutamic acid		C_7_ H_11_ N O_5_	1.256	188.0557	↓**	↑**	Arginine and proline metabolism
	Uric acid		C_5_ H_4_ N_4_ O_3_	0.838	167.0201	↑**	↓**	Purine metabolism
	2-Oxoglutaric acid		C_5_ H_6_ O_5_	0.841	145.0132	↓**	↑**	Arginine biosynthesis, TCA cycle, Alanine, aspartate, and glutamate metabolism, Butanoate metabolism, D-Glutamine, and D-glutamate metabolism
	L-Methionine		C_5_ H_11_ N O_2_ S	1.222	148.0427	↓**	↑**	Cysteine and methionine metabolism, Aminoacyl-tRNA biosynthesis
	2-Oxo-4-methylthiobutanoic acid		C_5_ H_8_ O_3_ S	2.403	147.011	↓**	↑**	Cysteine and methionine metabolism
	4-Oxoproline		C_5_ H_7_ N O_3_	0.835	128.0341	↓**	↑**	Glutathione metabolism
	Hypoxanthine		C_5_ H_4_ N_4_ O	1.22	135.0301	↓*	↑**	Purine metabolism
	Glyceric acid		C_3_ H_6_ O_4_	0.836	105.018	↓**	↑**	Glycine, serine and threonine metabolism, Glycerolipid metabolism, Glyoxylate and dicarboxylate metabolism, and Pentose phosphate pathway
	2-Hydroxybutyric acid		C_4_ H_8_ O_3_	1.277	103.0388		↑**	Propanoate metabolism
	2-Oxobutyrate		C_4_ H_6_ O_3_	0.827	101.0231	↓*	↑**	Cysteine and methionine metabolism, Valine, leucine, and isoleucine biosynthesis, Propanoate metabolism, Glycine, serine and threonine metabolism
	L-Lactic acid		C_3_ H_6_ O_3_	0.898	89.02303	↑*	↓**	Glycolysis / Gluconeogenesis, Pyruvate metabolism
ESI+	Thymine		C_5_ H_6_ N_2_ O_2_	1.819	127.0502		↑**	Pyrimidine metabolism
	L-Pyroglutamic acid		C_5_ H_7_ N O_3_	1.25	130.0499	↑**	↑**	Glutathione metabolism
	Nicotinic acid		C_6_ H_5_ N O_2_	1.232	124.0394	↓*	↑**	Nicotinate and nicotinamide metabolism
	Hypoxanthine		C_5_ H_4_ N4 O	0.851	137.0457	↓*	↑**	Purine metabolism
	Nicotinamide		C_6_ H_6_ N_2_ O	5.757	123.0554	↑**	↓**	Nicotinate and nicotinamide metabolism
	L-Methionine		C_5_ H_11_ N O_2_ S	0.87	150.0582	↓**	↑**	Cysteine and methionine metabolism, Aminoacyl-tRNA biosynthesis
	Adenosine		C_10_ H_13_ N_5_ O_4_	1.454	268.1035	↓**	↑**	Purine metabolism
	Inosine		C_10_ H_12_ N_4_ O_5_	1.473	269.0877		↑*	Purine metabolism
	β-Estradiol		C_18_ H_24_ O_2_	7.067	273.1844	↓**	↑**	Steroid hormone biosynthesis
	D-Proline		C_5_ H_9_ N O_2_	1.241	116.0709	↑**	↑**	Arginine and proline metabolism
	Dehydroepiandrosterone		C_19_ H_28_ O_2_	7.433	289.2156	↓**	↑**	Steroid hormone biosynthesis
	Uracil		C_4_ H_4_ N_2_ O_2_	1.24	113.0348	↓**	↑**	Pyrimidine metabolism, beta-Alanine metabolism, Pantothenate and CoA biosynthesis
	Arachidonic acid		C_20_ H_32_ O_2_	12.715	305.2467	↑**	↑**	Biosynthesis of unsaturated fatty acids, Arachidonic acid metabolism
	Phytosphingosine		C_18_ H_39_ N O_3_	7.678	318.2992	↓**	↓**	Sphingolipid metabolism
	3-HYDROXYPROPIONIC ACID		C_3_ H_6_ O_3_	0.807	91.03943	↑**	↓**	beta-Alanine metabolism, Propanoate metabolism

#### Metabolic pathway analysis

We utilized the Kyoto Encyclopedia of Genes and Genomes (KEGG) database to annotate potential biomarkers and identify the metabolic pathways they are associated with. The potential interrelationship among these biomarkers was represented by a network of metabolic pathways, part of which is depicted in Fig. 5. We conducted pathway enrichment analysis to identify the significantly altered pathways. Our results indicated that these biomarkers influence several metabolic pathways, including primary bile acid biosynthesis, biosynthesis of unsaturated fatty acids, tricarboxylic acid cycle, vitamin B6 metabolism, and purine metabolism, as summarized in Table 3. To further substantiate the LC-MS findings, ATP-binding cassette subfamily B member 11 (ABCB11), also referred to as the bile salt export pump, was selected for validation of the diminished cholic acid levels. ABCB11 holds a crucial role in expelling bile salts, inclusive of cholic acid, from hepatocytes into the bile canaliculi. Insufficient quantities of cholic acid might trigger a compensatory surge in ABCB11 expression or activity to enhance the transport mechanisms for other available bile salts [30, 31]. Thus, an immunohistochemical staining protocol was deployed to assess the ABCB11 expression levels in liver tissues. Observations indicated a prominently elevated expression of ABCB11 in mice subjected to high dosage treatments compared to the other groups, which served to further affirm the impact of decreased cholic acid content.

##### Primary bile acid biosynthesis

Primary bile acids are synthesized in the liver from cholesterol, and they play a key role in the digestion and absorption of fats, fat-soluble vitamins, and other nutrients in the intestine. Additionally, primary bile acids are known to be involved in the regulation of energy metabolism and have been linked to the development of hepatocellular carcinoma (HCC). Studies have shown that high concentrations of intrahepatic bile acids can lead to DNA damage in liver cells, which may increase the risk of oncogene mutations and contribute to the development of HCC. Therefore, changes in bile acid levels may serve as potential biomarkers for the early diagnosis and prognosis of HCC.

##### Biosynthesis of unsaturated fatty acids

Arachidonic acid (ARA) is an ω-6 unsaturated fatty acid that plays an important role in the liver as a phospholipid-bound structural lipid and is the direct precursor of prostaglandins E2 (PGE2), prostaglandin (PGI2), thromboxane A2 (TXA2), etc. [32] α-Linolenic acid (ALA) is an ω-3 unsaturated fatty acid, and studies have shown that this macronutrient exerts anti-inflammatory effects by producing oxylipins. In addition to anti-inflammatory mediators and antihypertensive effects, there is increasing evidence that ALA also plays a role in improving the phenotype of intestinal inflammatory diseases and preventing the occurrence of diseases such as colon cancer [33]. At the same time, ALA can interfere with the metabolic pathway of ARA and inhibit the direct production of PGI2, which indicates that the metabolic index ratio of ALA and ARA has a certain correlation with colon cancer and intestinal inflammation.

##### Purine metabolism

Purine is not only an important energy substance for the body, which provides energy for various physiological activities of cells but also forms nucleic acid molecules together with purine nucleotides and pyrimidine nucleotides, which are important genetic material in organisms. Purine nucleotide metabolism occurs primarily in the liver, kidney, and small intestine, so significant changes in purine metabolism can be identified as one of the potential factors for intestinal diseases and hyperuricemia [34].

##### Other metabolic pathways

Other metabolic pathways mainly include vitamin B6 metabolism, tryptophan metabolism, and arginine metabolism, which have a certain relationship with the occurrence and development of tumors. The Par index (the ratio of 4-pyridoxic acid to the sum of pyridoxal and pyridoxal-5’-phosphate) in vitamin B6 metabolism associated with inflammation is an important biomarker highly associated with lung cancer [35]. Tryptophan metabolites reduce cell proliferation and are potential biomarkers for a variety of tumors [36, 37]. Studies have shown that arginine metabolism can affect many important cellular components in the tumor microenvironment, including macrophages and T lymphocytes, leading to the suppression of immune surveillance. These findings support the use of arginine and arginase as important markers for tumor occurrence and development [38].

#### Metabolic pathway analysis of collaborative biomarkers

**Fig. 6:**
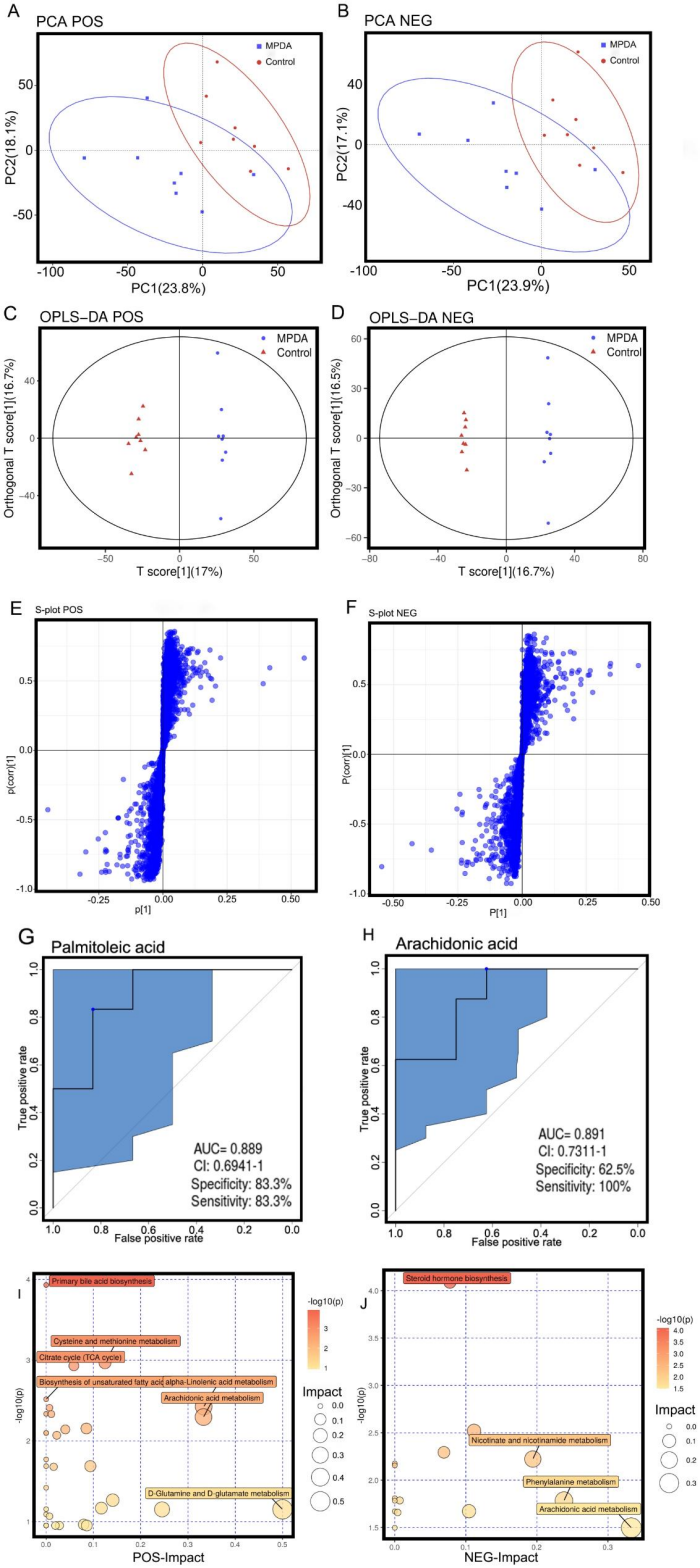
**(A, B)** The principal component analysis (PCA) map at positive and negative **(C, D)** The orthogonal projection to latent structures squares-discriminant analysis (OPLS-DA) model at positive and negative. **(E, F)** The S-plot map at positive and negative. **(G, H)** The ROC curves of palmitoleic acid and arachidonic acid **(I, J)** Volcano plot of the differential metabolites in stool at positive and negative

Furthermore, the KEGG database was used to identify the metabolic pathways affected by the biomarkers. The results revealed that the biomarkers influence several metabolic pathways, such as bile acid biosynthesis, citric acid cycle, arginine biosynthesis, and vitamin B6 metabolism, as displayed in Fig. 6I and J.

### Potential relationships between gut microbiota and fecal metabolites

The gut microbiota is important for maintaining health and can be disrupted for various reasons, leading to various diseases. Normal gut microbiota produces metabolites such as short-chain fatty acids, cholic acids, choline metabolites, indole derivatives, and vitamins. The gut microbiota is involved in regulating gene expression of metabolic and inflammatory pathways in the liver, and it plays a crucial role in the development of liver and gut disease through the gut-liver axis. The SCFA such as propionic acid and butyric acid released by Anaerostipes bacteria can inhibit fat deposition in a mouse model, the significant decrease in Anaerostipes bacteria observed in mice after MPDA treatment may increase the likelihood of developing liver-gut axis diseases such as nonalcoholic fatty liver disease (NASH). Therefore, Spearman’s correlation coefficient was used to analyze the relationship between host phenotypes, gut microbiota, and fecal metabolites in this study. The results suggest that there is a potential relationship between gut microbiota and fecal metabolites during MPDA intervention. For example, Lactobacillus_acidophilus was positively correlated with unsaturated fatty acids and indole substances and negatively correlated with bile acids and unsaturated fatty acids. This may be due to the regulation of gene expression of liver metabolism and inflammatory pathways by Lactobacillus_acidophilus through bile acids, leading to potential harm to the body.

In Table 3 of our research, a significant positive correlation was observed between bile acids and Gram-positive bacteria Firmicutes in the MPDA intervention group. Generally, a persistent decrease in total bile acids is beneficial for the growth of Gram-negative bacteria. These bacteria can produce pathogenic lipopolysaccharides, which is also one of the potential factors that can pose a threat to the organism.

## Discussion

As MPDA nanoparticles play important roles in tumor treatment, their metabolite toxicity study necessitates developing a better understanding of biosafety. In this regard, we systematically investigated the changes in bodyweight, routine blood parameters, organ index, pathology, fecal metabolites, and gut microbiota in mice after tail vein injection of MPDA nanoparticles at different concentrations. At the same time, potential biomarkers and differential flora in feces and serum were screened.

Compared with the control group, there were no significant abnormal changes in body weight, organ index or hematological parameters 7 days after treatment. Further study found that a high dose of MPDA had a significant effect on the gut-liver axis, mainly manifested in the presence of potential intestinal inflammation, apoptosis and oxidative stress. Moreover, a 16 S rRNA sequencing study showed that MPDA could destroy the homeostasis of intestinal flora, especially the increase in the abundance level of Lactobacillus acidophilus, and indirectly induce liver diseases and intestinal inflammation through bile acids and unsaturated fatty acids regulating gene expression of liver metabolism and inflammatory pathways.

However, this study had its own limitation. (1) Mice and humans have fundamental differences in their metabolism [39]. For example, mice generally have a higher metabolic rate than humans, meaning that drugs may be processed more quickly in mice and may require higher doses. Additionally, mice may have different enzymes and pathways for drug metabolism compared to humans, which can affect the way drugs are broken down and eliminated from the body [40]. However, we acknowledge the importance of understanding the potential translatability of these preclinical findings from mice to the clinical setting. Future studies could explore the pharmacokinetics and metabolism of MPDA in human subjects to further elucidate the potential clinical applications of this technology. (2) MPDA is a promising approach for targeted drug delivery in deep-seated tumors, as it integrates chemotherapy and photodynamic therapy. Recently, various MPDA-based nanoplatforms were developed and administrated by intravenous injection [41] to ensure sufficient circulation in the bloodstream and specific accumulation at the tumor site. Therefore, we chose the widely used tail vein injection method. However, we also recognize the potential benefits of oral administration of MPDA, which could potentially simplify drug delivery and enhance patient compliance. Thus, testing the toxicity of oral MPDA in ICR mice in the future is considerable.

## Conclusion

In conclusion, our findings suggest that a high-dose application of MPDA (78.57 mg/kg) may lead to disruptions in the gut microbiota, thereby intensifying intestinal inflammation and oxidative stress injury. Hence, careful dosage management is essential not only for effective tumor treatment, but also for the concurrent monitoring of intestinal conditions. This study represents the first comprehensive exploration into the systematic toxicity of MPDA-based nanoparticles, providing critical data that bridges the gap between research and clinical application.

### Electronic supplementary material

Below is the link to the electronic supplementary material.

Supplementary Material 1

## Data Availability

The datasets generated and analyzed during this study are available from the corresponding authors on reasonable request.
