# Supplementary material for: The subacute toxicity and underlying mechanisms of biomimetic mesoporous polydopamine nanoparticles
Source: Part Fibre Toxicol. 2023 Oct 8;20:38. doi: 10.1186/s12989-023-00548-4 (PMC10560437; doi:10.1186/s12989-023-00548-4)
Supplement: Supplementary file 1 — Supplementary Material 1 [file 12989_2023_548_MOESM1_ESM.docx]

**The subacute toxicity and underlying mechanisms of biomimetic mesoporous polydopamine nanoparticles**

Bang-Yao Chen**^#,1^**, Si-Ying Hong**^#,1^**, Han-Min Wang**^#,1^**, Yi Shi**^2^**, Peng Wang**^2^**, Xiao-Juan Wang**^3^**, Qian-Yang Jiang**^1^**, Ke-Da Yang**^*,1^**, Wei Chen**^*,2^**, Xiao-Ling Xu**^*,1^**

**^1^**Shulan International Medical College, Zhejiang Shuren University, Hangzhou 310015, PR China;

**^2^**ICU, Longhua Hospital Affiliated to Shanghai University of Traditional Chinese Medicine, Shanghai, 200032, PR China;

**^3^**Department of Clinical Pharmacy, The First Affiliated Hospital, School of Medicine, Zhejiang University, 79 Qingchun Road, Hangzhou 310003, PR China.

**^#^**These authors contributed equally.

Correspondence to:

Dr. X. L. Xu (Shulan International Medical College, Zhejiang Shuren University), 8 Shuren Street, Hangzhou, 310015, China. E-mail: [ziyao1988@zju.edu.cn](mailto:ziyao1988@zju.edu.cn)

Dr. W. Chen (ICU, Longhua Hospital Affiliated to Shanghai University of Traditional Chinese Medicine), 725 South WanPing Road, Shanghai, 200032, China. Tel: +86-021-64385700-3522. E-mail: cwdoctor@shutcm.edu.cn;

Dr. K. D. Yang (Shulan International Medical College, Zhejiang Shuren University), 8 Shuren Street, Hangzhou, 310015, China. E-mail: [kdyang@zjsru.edu.cn](mailto:kdyang@zjsru.edu.cn)

Figure S1 The changes of particle size at day 1, 3, 5 and 7.

Figure S2 The changes of drug encapsulation efficiency at day 1, 3, 5 and 7.


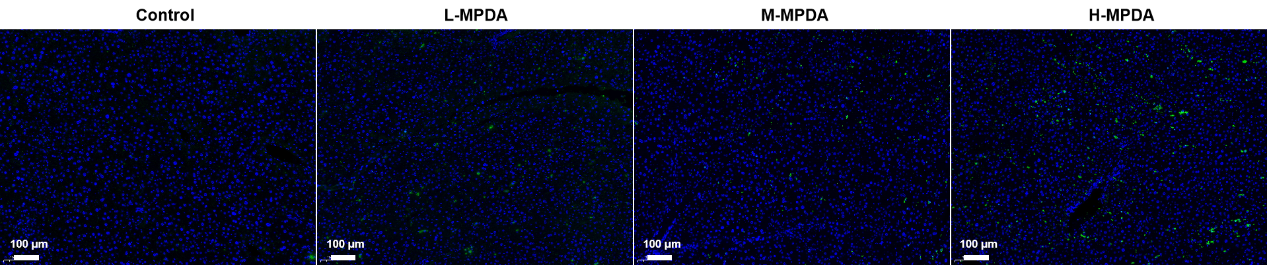


Figure S3 The expression of ATP-binding cassette subfamily B member 11 (ABCB11) in the liver tissues.
